# Temperature during conidiation affects stress tolerance, pigmentation, and trypacidin accumulation in the conidia of the airborne pathogen *Aspergillus fumigatus*

**DOI:** 10.1371/journal.pone.0177050

**Published:** 2017-05-09

**Authors:** Daisuke Hagiwara, Kanae Sakai, Satoshi Suzuki, Myco Umemura, Toshihiko Nogawa, Naoki Kato, Hiroyuki Osada, Akira Watanabe, Susumu Kawamoto, Tohru Gonoi, Katsuhiko Kamei

**Affiliations:** 1 Medical Mycology Research Center (MMRC), Chiba University, 1-8-1 Inohana, Chuo-ku, Chiba, Japan; 2 National Food Research Institute (NFRI), 2-1-12 Kan-nondai, Tsukuba, Ibaraki, Japan; 3 National Institute of Advanced Industrial Science and Technology (AIST), 17-2-1 Higashi-Nijo, Tsukisamu, Toyohira-ku, Sapporo, Japan; 4 RIKEN Center for Sustainable Resource Science, Wako, Saitama, Japan; Leibniz-Institut fur Naturstoff-Forschung und Infektionsbiologie eV Hans-Knoll-Institut, GERMANY

## Abstract

Asexual spores (conidia) are reproductive structures that play a crucial role in fungal distribution and survival. As fungal conidia are, in most cases, etiological agents of plant diseases and fungal lung disease, their stress resistance and interaction with their hosts have drawn increasing attention. In the present study, we investigated whether environmental temperature during conidiation affects the stress tolerance of the conidia of the human pathogenic fungus *Aspergillus fumigatus*. Conidia from a 25°C culture showed a lower tolerance to heat (60°C) and oxidative (H_2_O_2_) stresses and a marked resistance to ultraviolet radiation exposure, compared with those produced at 37 and 45°C. The accumulation of trehalose was lower in the conidia from the 25°C culture. Furthermore, the conidia from the 25°C culture showed darker pigmentation and increased transcripts of dihydroxynaphthalene (DHN)-melanin biosynthesis-related genes (i.e., *pksP*, *arp1*, and *arp2*). An RNA-sequencing analysis revealed that the transcription level of the trypacidin (*tpc*) gene cluster, which contains 13 genes, was sharply and coordinately activated in the conidia from the 25°C culture. Accordingly, trypacidin was abundant in the conidia from the 25°C culture, whereas there was little trypacidin in the conidia from the 37°C culture. Taken together, these data show that the environmental temperature during conidiation affects conidial properties such as stress tolerance, pigmentation, and mycotoxin accumulation. To enhance our knowledge, we further explored the temperature-dependent production of DHN-melanin and trypacidin in clinical *A*. *fumigatus* isolates. Some of the isolates showed temperature-independent production of DHN-melanin and/or trypacidin, indicating that the conidia-associated secondary metabolisms differed among the isolates.

## Introduction

Filamentous fungi vigorously produce asexual spores (conidia) under appropriate conditions. Conidia are reproductive structures that play a crucial role in the distribution and survival of fungi [1]. In general, *Aspergillus* conidia are relatively stress-resistant cells that survive environmental stresses such as drought, high temperatures, and ultraviolet (UV) irradiation [2,3]. Fungi protect themselves from such abiotic stresses by accumulating compatible solutes. Mannitol and trehalose are the most prevalent solute sugars that accumulate in conidia. While mannitol is the most abundant solute in *Aspergillus oryzae* and *Aspergillus niger* conidia, trehalose is the major compatible solute that accumulates in *Aspergillus fumigatus* conidia [4–6], The absence of the compatible solutes reduces the heat resistance and/or longevity of *A*. *niger* and *Aspergillus nidulans* conidia [2,7].

*A*. *fumigatus* is the causative agent of aspergillosis, which encompasses several types of infection, including life-threatening invasive aspergillosis. Conidia are the main infectious agents, and they are inhaled daily because they are ubiquitous in the environment [8,9]. The conidia of *A*. *fumigatus* have dihydroxynaphthalene (DHN)-melanin in their cell wall, which is thought to provide structural rigidity and protection from environmental stresses, such as UV irradiation and heat [10]. A genetic study highlighted the crucial role of DHN-melanin in *A*. *fumigatus* virulence, because the virulence of a polyketide synthase (PKS) mutant, *pksP*, which fails to produce the PKS that is responsible for DHN-melanin synthesis, was attenuated in a murine infection model of disseminated aspergillosis [11]. Furthermore, DHN-melanin protects conidia against phagocytic killing by inhibiting the acidification of phagolysosomes [12,13]. Additionally, pigment-less, white conidia of a *pksP* mutant were ingested more frequently compared with wild-type (WT) conidia by the soil-living amoeba *Dictyostelium discoideum* [14]. Thus, DHN-melanin not only plays an essential role in infections of mammalian hosts but also contributes to avoiding predation by amoeba. Different types of melanin (Asp-melanin) on the conidia of *Aspergillus terreus* also inhibit phagocytosis by *D*. *discoideum* [15], which supports the importance of melanin for fungal conidia to survive in their environments.

*A*. *fumigatus* is also a major airborne allergen, and it possesses 22 known allergenic proteins. Temperature impacts the allergenicity and viability of *A*. *fumigatus* conidia [16]. An increased level of the Asp f1 major allergen was found in conidia collected from cultures grown at 17°C, compared with those grown at 32°C. Environmental conditions during conidiation affect the physiological properties, such as morphology, germination kinetics, and pathogenicity, of spores from other fungi [17–20]. In addition, entomopathogenic fungi have demonstrated that virulence toward insect hosts, and conidial tolerance to heat and UV radiation, are greatly influenced by environmental conditions such as the medium, water activity, light illumination, and oxygen concentration, during conidia production [21,22]. Thus, environmental conditions appear to be key factors that affect fungal conidial properties; however, little is known about the impacts of temperature on the properties of the conidia of *Aspergillus* species, including the medically important pathogenic fungus *A*. *fumigatus*.

Here, we investigated the effects of temperature during conidia production on the stress resistance of *A*. *fumigatus* conidia. Based on a comparative transcriptome analysis, we found that conidia from a 25°C culture accumulated greater amounts of DHN-melanin and the cytotoxic secondary metabolite trypacidin, compared with those from a 37°C culture.

## Results

### Conidia harvested from cultures grown at different temperatures show divergent stress resistances

**Fig 1:**
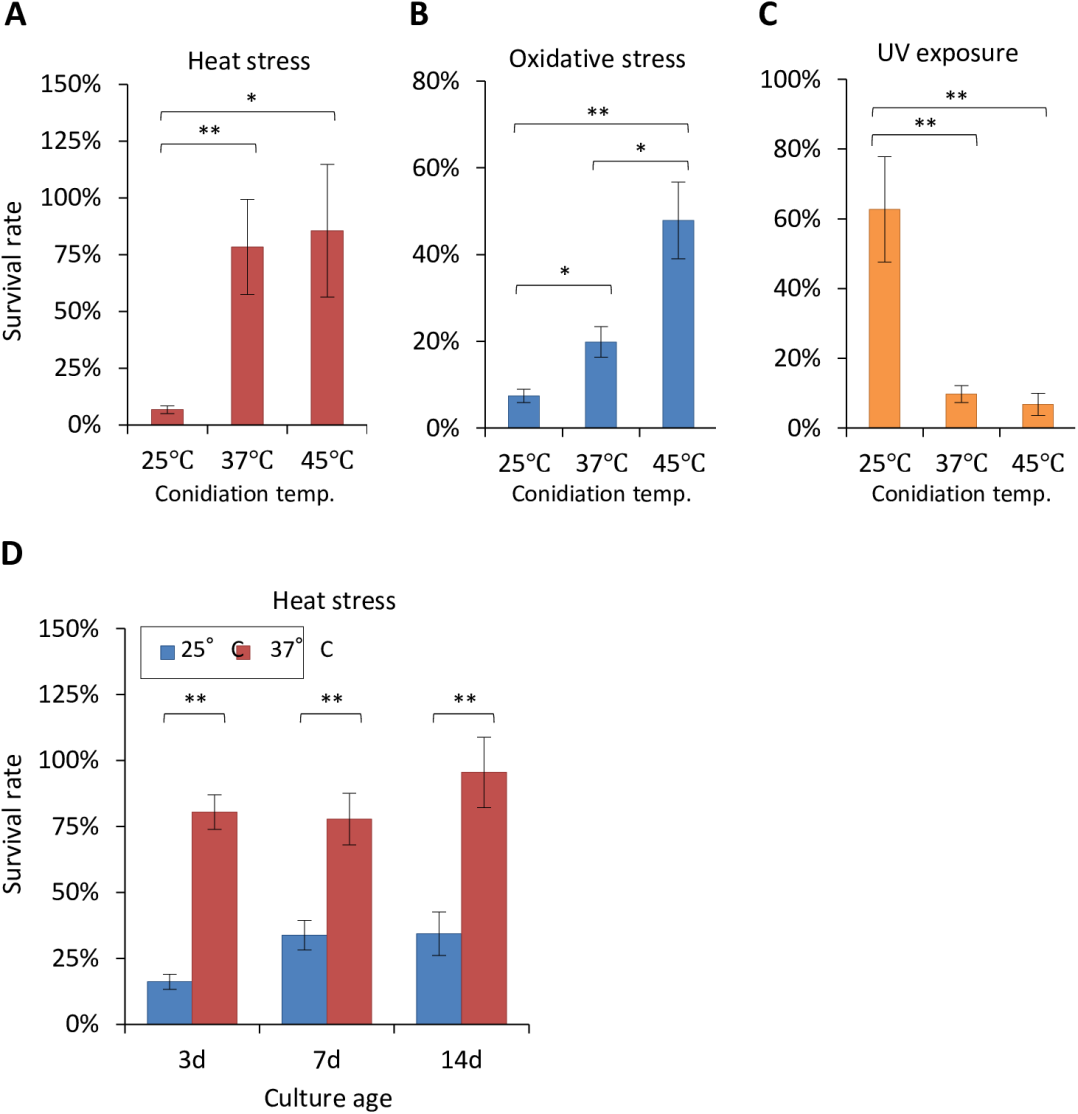
Comparison of conidial stress resistance. Conidia of *A*. *fumigatus* strain Af293 were harvested after 7 d of growth on PDA at 25, 37, and 45°C. The survival rates upon heat stress (60°C, 15 min) (A), oxidative stress (200 mM hydrogen peroxide, 15 min) (B), and UV stress (573 J m^−2^) (C) are shown. The survival rate (%) was calculated by dividing the number of colony-forming units (CFU) following the stress treatment by the number of CFU obtained in the absence of treatment. (D) Upon heat stress (60°C, 15 min) the survival rates of the conidia harvested after 3, 7, and 14 d of growth at 25 and 37°C were shown. Each sample was tested in 3 or 4 technical replicates. Error bars represent the standard deviation. Experiments were repeated twice, and representative experiments are shown. Statistical analysis (Student’s *t*-test) was performed, and significant differences were indicated as an asterisk (**:*p*<0.01; *:*p*<0.05).

To examine the impact of temperature on conidial physiology, we cultivated the *A*. *fumigatus* Af293 strain on potato dextrose agar (PDA) for 7 d at 25, 37, and 45°C, after which the conidia were collected. We investigated how the conidia tolerated short bursts of heat stress (60°C for 15 min), oxidative stress [200 mM hydrogen peroxide (H_2_O_2_) for 15 min], and UV stress (9.55 Wm^−2^ of UV light for 1 min) that are encountered by fungal conidia in nature. The conidia from the 25°C culture showed a significantly greater sensitivity to heat stress, compared with those from the 37 and 45°C cultures (Fig 1A). Upon H_2_O_2_ treatment (as an oxidative stressor), the conidia from the 25°C culture were more sensitive than those from the 37°C culture, whereas the conidia from the 45°C culture showed a greater stress tolerance (Fig 1B). In contrast, the conidia from the 25°C culture showed a marked tolerance to UV irradiation (Fig 1C). To rule out the possibility that the conidial maturation levels affected the differences in phenotypes, we compared the heat-stress tolerance of the conidia harvested from the 25 and 37°C cultures in a time-dependent manner (Fig 1D). The conidia from the 25°C culture were more sensitive to heat stress than those from the 37°C at all of the culture ages (3d, 7d, and 14d). Thus, the conidiation temperature affected the stress tolerance of *A*. *fumigatus* conidia.

### Reduced trehalose levels in conidia from a 25°C culture

**Fig 2:**
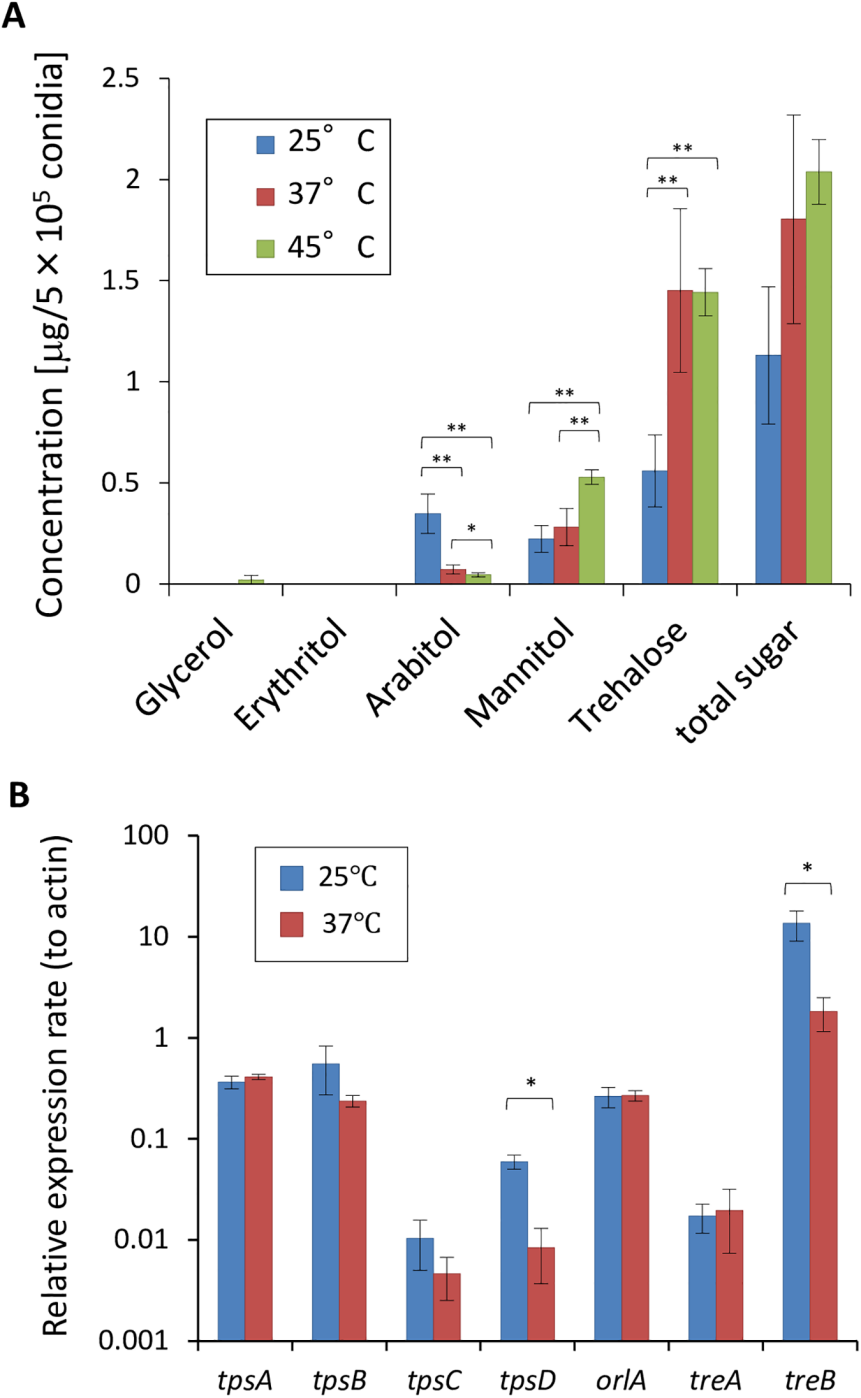
Polyols and trehalose contents of conidia. (A) Polyols and trehalose were extracted from conidia and analyzed by high-performance liquid chromatography (HPLC). Error bars represent the standard deviation based on six independent replicates. (B) Expression analysis of trehalose metabolism related genes in the conidia from the 25 and 37°C cultures. RNA was extracted from Af293 conidia that were produced at the indicated temperature. The expression of *tpsA-D*, *orlA*, and *treA*,*B* was determined by quantitative real-time polymerase chain reaction (qRT-PCR). Each sample was tested in three biological replicates. Error bars represent the standard deviation. Statistical analysis (Student’s *t*-test) was performed, and significant differences were indicated as an asterisk (**:*p*<0.01; *:*p*<0.05).

Trehalose is the major compatible solute in *A*. *fumigatus* conidia that protects against environmental stresses, including heat and oxidation [5,23]. Thus, we examined the amounts of carbohydrates that accumulated in conidia from cultures grown at 25, 37, and 45°C. The conidia from the 25°C culture exhibited a lower level of trehalose than those from the 37 and 45°C cultures (Fig 2A). This reduction was assumed to cause the heat and oxidative stress-labile phenotypes of the conidia from the 25°C culture. Notably, the conidia from the 25°C culture accumulated an abundant amount of arabitol, whereas the conidia grown at the other temperatures exhibited only low levels of arabitol. This may be a compensating metabolic effect, but its role in the aforementioned stress tolerance remains unknown.

To understand the mechanism underlying the lower trehalose level in the conidia from the 25°C culture, transcript levels of trehalose metabolism-related genes, including the trehalose-6-phosphate synthases *tpsA* to *tpsD*, the trehalose-6-phosphate phosphatase *orlA*, and the trehalases *treA*, and *treB* were investigated (Fig 2B). The conidia from the 25°C culture accumulated greater amounts of *treB* transcripts compared with those from the 37°C culture, suggesting the more efficient degradation of trehalose in the conidia. However, the higher transcript levels of *tpsD* in the conidia from 25°C culture were shown, which might promote trehalose production.

### DHN-melanin accumulates at high levels in conidia from a 25°C culture

**Fig 3:**
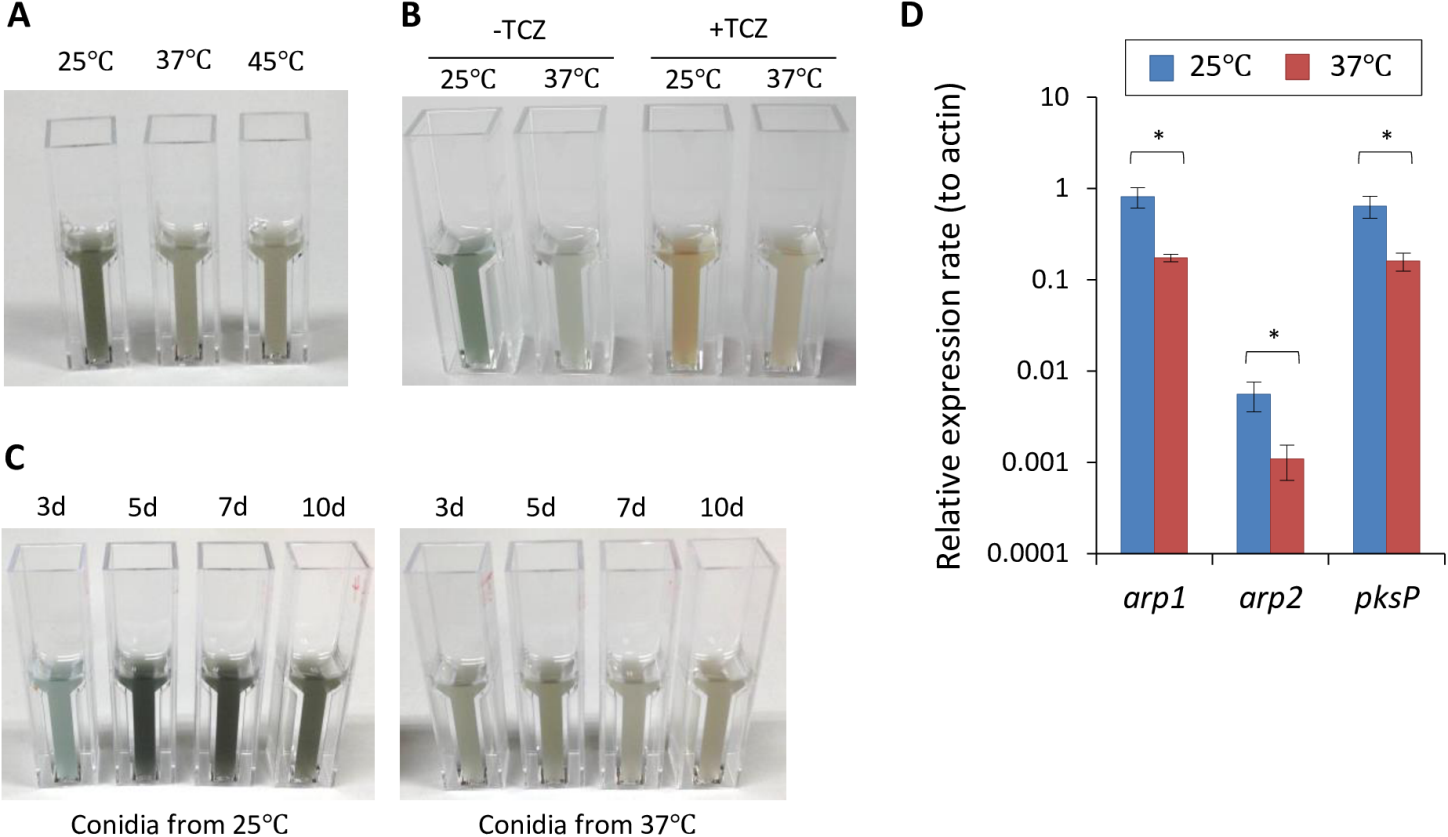
Comparison of conidial pigmentation. (A) Conidia of *A*. *fumigatus* strain Af293 were harvested from 25, 37, and 45°C cultures. The conidial suspensions were diluted to 1x10^8^ conidia mL^−1^ and photographed. (B) Conidia were harvested after cultivation in the presence or absence of tricyclazole (TCZ). The conidial suspensions were diluted to 5x10^7^ conidia mL^−1^ and photographed. (C) The conidia were harvested from 25 and 37°C cultures at days 3, 5, 7, and 10. The conidial suspensions were diluted to 1x10^8^ conidia mL^−1^ and photographed. (D) Expression analysis of the DHN-melanin biosynthesis pathway in the conidia from the 25 and 37°C cultures. RNA was purified from Af293 conidia that were produced at the indicated temperature. The relative expression ratio of *arp1*, *arp2*, and *pksP* to actin gene was determined by qRT-PCR. Each sample was tested in three biological replicates. Error bars represent the standard deviation. Statistical analysis (Student’s *t*-test) was performed, and significant differences were indicated as an asterisk (*:*p*<0.05).

We compared the structural properties of conidia isolated from cultures that were grown at different temperatures. Under microscopic observation, the size and form of the conidia were comparable among all of the cultures (data not shown). However, the pigmentation level differed, as the conidia from the 25°C culture exhibited a darker, greenish-gray color (Fig 3A), which indicates the presence of DHN-melanin on their surface. To determine whether the pigmentation in the conidia from the 25°C culture was caused by DHN-melanin, we used tricyclazole (TCZ), a specific inhibitor of DHN-melanin biosynthesis [24]. In the presence of TCZ, the conidia were brown, and the 25°C culture was a darker brown than the 37°C culture (Fig 3B). This suggests that a lower temperature during conidiation promoted a higher level of DHN-melanin accumulation in the conidia. We also confirmed pigmentation levels of the conidia in a series of culture ages, with the conidia from 5, 7, and 10 d of growth at 25°C revealing dense pigmentation compared with those harvested from the culture at 37°C (Fig 3C). This indicated that culture age did not affect the pigmentation levels but the temperature did.

DHN-melanin is synthesized through a pathway that includes the proteins PksP, Ayg1, Arp2, Arp1, Abr1, and Abr2 during conidiogenesis and conidial maturation [10]. Thus, we investigated the transcriptional levels of the corresponding genes between conidia that were grown at 25 and 37°C. The transcripts of *pksP*, which encodes a PKS that is involved in the first step of the pathway, were four times more abundant in the conidia from the 25°C culture than in those from the 37°C culture (Fig 3D). *arp1* and *arp2* were also expressed at higher levels in the conidia from the 25°C culture, which confirmed that the DHN-melanin biosynthesis pathway was highly expressed in conidia grown at 25°C, and this was likely responsible for the abundance of DHN-melanin on their surfaces.

### Comparing transcriptomes among conidia produced at different temperatures

To gain a broader insight into the physiological impact of temperature on conidia, we conducted an RNA sequencing analysis to generate genome-wide transcriptional profiles. The RNAs were extracted from resting conidia that were collected from cultures that were grown on PDA for 7 d at 25, 37, and 45°C (see the Material and Methods). The whole transcriptome data is provided in S3 Table. Transcripts of 8,953 genes were detected in the conidia from the 25, 37, and 45°C cultures. Among them, 492 and 696 genes showed greater than 4-fold increases in transcript levels in the conidia from the 25 and 45°C cultures, respectively, compared with those from the 37°C culture. Among these, the genes with enriched transcripts [showing more than 25% of mean fragments per kilobase of transcript per million mapped reads (FPKM) value and >10-fold increased] are listed in Table 1. The genes whose transcripts were up-regulated in the conidia from the 45°C culture included those encoding a non-ribosomal peptide synthase (NRPS)-like enzyme (AFUA_8G01640) and a GPI-anchored cell wall organization protein Ecm33. On the contrary, the increased expression levels of *rodA*, *ayg1*, and *conF*, as well as trypacidin biosynthesis genes were found in the conidia from the 25°C culture. The transcript levels of the allergen-related *Asp f* genes are listed in S2 Table. In total, 11, 10, and 10 out of the 22 *Asp f* genes showed higher FPKM values than the mean FPKMs (113.0, 103.3, and 93.7) in the conidia from the 25, 37, and 45°C cultures, respectively. However, no obvious trend in the temperature-dependent transcriptional changes was found.

**Table 1:** FPKM data for the genes that were enriched at different conidiation temperatures.

	FPKM			Ratio			
Gene ID	Conidia from 25°C	Conidia from 37°C	Conidia from 45°C	25°C/37°C	45°C/37°C	Gene name	Annotation
**Enriched in 45°C conidia**							
AFUA_7G00150	0.6	1.6	**48.0**	0.37	**30.24**	*nscC*	FAD-dependent monooxygenase, putative
AFUA_1G01080	2.4	1.7	**42.3**	1.40	**25.06**		conserved hypothetical protein
AFUA_5G09020	4.8	2.7	**49.3**	1.73	**17.96**	*wsc3*	ER membrane protein Wsc4, putative
AFUA_3G13570	10.7	2.7	**37.7**	4.00	**14.08**		conserved hypothetical protein
AFUA_7G01470	5.4	4.4	**61.0**	1.24	**14.02**		integral membrane protein, Mpv17/PMP22 family, putative
AFUA_7G01210	11.6	2.1	**28.8**	5.60	**13.87**		conserved hypothetical protein
AFUA_1G05890	13.7	2.1	**28.5**	6.54	**13.59**		hypothetical protein
AFUA_8G01640	0.5	180.0	**2370.4**	0.00	**13.17**		NRPS-like enzyme, putative
AFUA_4G06820	26.4	6.5	**77.2**	4.06	**11.85**	*ecm33*	GPI-anchored cell wall organization protein Ecm33
AFUA_4G06670	3.6	3.4	**39.1**	1.07	**11.65**	*aspf7*	allergen Asp F7
AFUA_1G06200	5.5	4.7	**54.0**	1.17	**11.54**		Mn2+ homeostasis protein (Per1), putative
AFUA_3G02640	3.6	3.5	**39.7**	1.05	**11.47**		nucleoside-diphosphate-sugar epimerase family protein
**Enriched in 25°C conidia**							
AFUA_4G00640	**56.5**	0.4	1.9	**126.97**	4.25		macrophomate synthase, putative
AFUA_1G01980	**3039.2**	31.0	34.3	**97.92**	1.11		IgE binding protein, putative
AFUA_5G07420	**98.9**	2.2	3.1	**44.35**	1.38		hypothetical protein
AFUA_5G00660	**502.9**	11.6	1.4	**43.21**	0.12		hypothetical protein
AFUA_4G14580	**1132.4**	28.0	12.3	**40.48**	0.44	*tpcA*	O-methyltransferase, putative
AFUA_4G14490	**799.4**	20.4	4.3	**39.10**	0.21	*tpcJ*	extracellular dihydrogeodin oxidase/laccase, putative
AFUA_4G14570	**1091.1**	28.3	7.3	**38.52**	0.26	*tpcB*	metallo-beta-lactamase domain protein, putative
AFUA_5G09030	**35.9**	1.0	0.8	**36.65**	0.85		F-box domain and ankyrin repeat protein
AFUA_4G14520	**509.1**	14.3	11.7	**35.67**	0.82	*tpcG*	toxin biosynthesis protein, putative
AFUA_4G14460	**2001.8**	58.5	21.4	**34.24**	0.37	*tpcM*	conserved hypothetical protein
AFUA_4G14510	**3137.1**	95.0	23.8	**33.01**	0.25	*tpcH*	hypothetical protein
AFUA_8G06890	**101.5**	3.2	7.3	**32.02**	2.29		extracellular exo-polygalacturonase, putative
AFUA_4G14480	**1253.4**	40.1	7.5	**31.26**	0.19	*tpcL*	conserved hypothetical protein
AFUA_4G14470	**1073.0**	34.5	8.5	**31.08**	0.25	*tpcK*	hypothetical protein
AFUA_5G01260	**572.4**	19.4	6.0	**29.54**	0.31		ankyrin repeat protein
AFUA_4G14560	**270.6**	9.3	4.5	**29.01**	0.48	*tpcC*	polyketide synthase, putative
AFUA_4G14500	**454.7**	15.7	3.2	**28.91**	0.20	*tpcI*	conserved hypothetical protein
AFUA_2G12690	**118.1**	4.6	3.9	**25.68**	0.85		hypothetical protein
AFUA_2G00140	**33.0**	1.4	2.8	**24.41**	2.06		FAD monooxygenase, putative
AFUA_7G06820	**99.8**	4.2	1.4	**23.91**	0.33		galactose oxidase, putative
AFUA_2G00150	**96.2**	4.2	2.4	**23.03**	0.57		catecholamine-O-methyltransferase, putative
AFUA_1G05910	**2155.0**	99.0	85.0	**21.78**	0.86		conserved hypothetical protein
AFUA_4G14530	**1173.8**	56.9	64.5	**20.62**	1.13	*tpcF*	glutathione S-transferase Ure2-like, putative
AFUA_4G14540	**151.9**	7.9	15.3	**19.33**	1.95	*tpcE*	C6 transcription factor (AflR), putative
AFUA_4G14550	**434.8**	22.5	51.6	**19.29**	2.29	*tpcD*	toxin biosynthesis regulatory protein AflJ, putative
AFUA_8G01210	**75.6**	4.4	1.2	**17.32**	0.28		enoyl-CoA hydratase/isomerase family protein
AFUA_3G14260	**2943.7**	174.2	125.2	**16.90**	0.72		mismatched base pair and cruciform DNA recognition protein, putative
AFUA_7G05100	**64.8**	3.9	15.5	**16.69**	3.98		hexose transporter protein
AFUA_3G13130	**87.4**	5.3	11.4	**16.60**	2.17		HHE domain protein
AFUA_4G03010	**930.8**	56.6	57.9	**16.44**	1.02		conserved hypothetical protein
AFUA_7G06840	**1858.2**	114.1	53.6	**16.29**	0.47		class III aminotransferase, putative
AFUA_1G11560	**71.4**	4.7	15.9	**15.23**	3.40		4-hydroxyphenylpyruvate dioxygenase, putative
AFUA_6G14510	**138.1**	9.6	16.5	**14.44**	1.72		monooxygenase, putative
AFUA_6G01980	**41.2**	2.9	14.9	**14.40**	5.22		haemolysin-III family protein
AFUA_6G09570	**403.6**	28.3	23.2	**14.24**	0.82		conserved hypothetical protein
AFUA_5G09580	**1436.6**	103.9	108.3	**13.82**	1.04	*rodA*	conidial hydrophobin Hyp1/RodA
AFUA_7G06830	**240.8**	17.4	11.6	**13.82**	0.66		MFS tranporter, putative
AFUA_8G07260	**42.8**	3.1	3.1	**13.69**	0.99		conserved hypothetical protein
AFUA_8G04920	**201.1**	14.9	8.2	**13.49**	0.55		LEA domain protein
AFUA_7G00950	**285.3**	22.1	26.7	**12.89**	1.21		MFS monosaccharide transporter, putative
AFUA_5G00145	**101.3**	8.1	2.5	**12.48**	0.31		conserved hypothetical protein
AFUA_4G00730	**3748.9**	308.7	61.8	**12.14**	0.20		HHE domain protein
AFUA_3G14750	**31.9**	2.6	1.3	**12.08**	0.48		fungal specific transcription factor, putative
AFUA_2G11870	**116.2**	10.0	21.4	**11.59**	2.13	*kre6*	beta-1,6 glucan synthetase (Kre6), putative
AFUA_7G08320	**48.8**	4.2	10.8	**11.51**	2.55		heat shock transcription factor, putative
AFUA_7G01720	**647.0**	56.9	95.9	**11.36**	1.68		3-hydroxymethyl-3-methylglutaryl-Coenzyme A lyase
AFUA_2G17550	**208.0**	18.5	6.2	**11.24**	0.34	*ayg1*	conidial pigment biosynthesis protein Ayg1
AFUA_1G16960	**2413.7**	215.9	310.3	**11.18**	1.44		conserved hypothetical protein
AFUA_4G08170	**979.3**	92.1	68.5	**10.64**	0.74	*uga2*	succinate-semialdehyde dehydrogenase Uga2, putative
AFUA_1G13800	**507.9**	48.3	23.4	**10.52**	0.48		MFS multidrug transporter, putative
AFUA_1G05920	**69.5**	6.8	10.8	**10.20**	1.59		conserved hypothetical protein
AFUA_6G08780	**128.8**	12.7	22.3	**10.11**	1.75		proline utilization protein PrnX, putative
AFUA_4G03615	**7668.7**	764.5	266.3	**10.03**	0.35	*conF*	conidiation protein Con-6, putative

### Temperature-dependent synthesis of trypacidin in conidia

**Fig 4:**
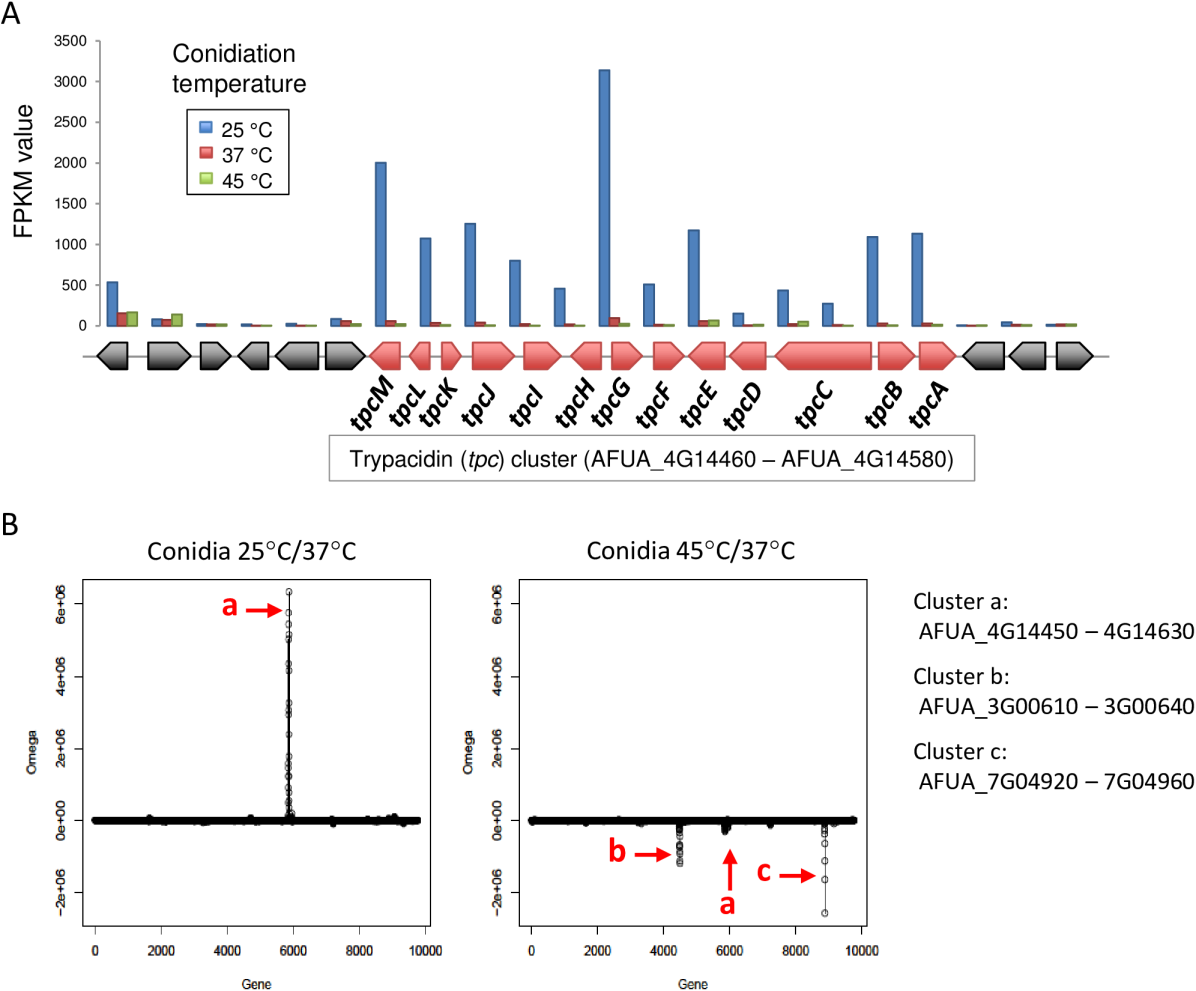
The trypacidin (*tpc*) cluster is induced in conidia at a low temperature. (A) Using data from the RNA sequencing analysis, expression values (FPKM) for the genes in the *tpc* cluster were depicted. The arrows shown below the graph indicate the direction and relative length of the genes. (B) The MIDDAS-M analysis revealed that the expression of clustered genes is coordinately altered in the conidia from 25 and 45°C cultures, compared with those from a 37°C culture.

According to Sanchez et al. [25] and the *Aspergillus* Genome Database (AspGD) annotations, *A*. *fumigatus* possesses 18 (putative or validated) NRPS and NRPS-like genes, 15 PKS and PKS-like genes, and one PKS-NRPS hybrid gene. The expression profiles of the NRPS and PKS genes are listed in Table 2. Among these, one NRPS-like gene (AFUA_8G01640) and two PKS genes [*pksP* and *tpcC* (also called *tynC*)] were found to be relatively highly expressed in the conidia. AFUA_8G01640 was more highly expressed in the conidia that were grown at 45°C, whereas *pksP* and *tpcC/tynC*, which are responsible for DHN-melanin and trypacidin biosynthesis, respectively, were more highly expressed at 25°C. The trypacidin biosynthesis (*tpc/tyn*) gene cluster was recently reported to comprise 13 contiguous genes (*tpcA/tynA* to *tpcM/tynM*) [26,27]. Indeed, the present RNA sequencing analysis showed that the expression of the *tpc* genes was coordinately regulated in a temperature-dependent manner, with clear borders on both ends of the gene cluster (Fig 4A).

**Table 2:** FPKM data for secondary metabolism genes.

	FPKM			Ratio			
Gene ID	Conidia from 25°C	Conidia from 37°C	Conidia from 45°C	25°C/37°C	45°C/37°C	Gene	Predicted or actual products
**NRPS or NRPS-like**							
AFUA_1G10380	5.1	0.8	0.9	6.01	1.06	*pes1/nrps1*	Fumigaclavine C
AFUA_1G17200	3.4	1.8	4.0	1.91	2.23	*sidC*	Ferricrocin, hydroxyferricrocin
AFUA_3G02670	17.9	6.5	27.0	2.77	4.17		
AFUA_3G03350	0.3	0.2	0.9	1.40	3.61	*sidE*	
AFUA_3G03420	9.7	0.4	0.1	25.99	0.14	*sidD*	Fusarinine C, triacetylfusarinine C
AFUA_3G12920	0.1	0.9	1.7	0.12	1.93	*hasD*	Hexadehydroastechrome
AFUA_3G13730	3.7	2.6	1.1	1.40	0.42	*pesG*	
AFUA_3G15270	0.1	0.0	0.1	-	-	*pesH*	
AFUA_5G10120	5.9	6.1	18.8	0.96	3.07		
AFUA_5G12730	43.2	28.4	12.0	1.52	0.42	*pes3*	
AFUA_6G03480	0.4	0.4	1.5	0.93	3.40		
AFUA_6G08560	5.2	4.1	15.7	1.27	3.81		
AFUA_6G09610	2.1	0.3	2.0	6.22	5.95	*pesJ*	
AFUA_6G09660	1.2	0.4	5.4	2.80	12.74	*gliP*	Gliotoxin
AFUA_6G12050	11.0	3.2	7.8	3.38	2.40	*fmgC*	Fumiquinazolines
AFUA_6G12080	2.0	0.7	0.9	2.76	1.31	*fmgA*	Fumiquinazolines
AFUA_8G00170	0.3	0.1	0.8	5.60	13.59	*ftmA*	FumitremorginS
AFUA_8G01640	0.5	180.0	2370.4	0.00	13.17		
**PKS or PKS-like**							
AFUA_1G01010	0.0	0.1	0.4	0.00	2.97		
AFUA_1G17740	1.2	1.9	9.3	0.64	4.93		
AFUA_2G01290	6.7	6.0	8.8	1.13	1.47		
AFUA_2G17600	207.3	74.9	18.0	2.77	0.24	*pksP*	DHN-melanin
AFUA_3G01410	8.4	8.1	20.0	1.03	2.46		
AFUA_3G02530	5.5	5.9	7.8	0.93	1.33		
AFUA_3G02570	7.2	6.5	9.2	1.10	1.41		
AFUA_3G14700	0.3	0.1	0.5	3.27	5.10		
AFUA_4G00210	2.4	1.6	4.3	1.49	2.70	*encA*	Endochrocin
AFUA_4G14560	270.6	9.3	4.5	29.01	0.48	*tpcC*	
AFUA_6G13930	0.3	1.2	1.5	0.25	1.27		
AFUA_7G00160	3.3	11.4	34.6	0.29	3.04	*nscA/fccA*	Neosartoricin/fumicycline A
AFUA_8G00370	0.5	0.3	0.6	1.71	1.98	*fma-PKS*	Fumagilin
AFUA_8G00490	0.0	0.1	4.7	0.00	36.52		
AFUA_8G02350	2.0	3.0	6.3	0.69	2.12		
**PKS-NRPS hybrid**							
AFUA_8G00540	0.5	0.2	1.0	2.99	6.46	*psoA*	Pseurotin

We also performed a motif-independent, *de novo* detection of secondary metabolite gene clusters using the MIDDAS-M program [28]. In addition to the *tpc* cluster, this analysis revealed that the expression levels of two additional sets of genes were coordinately regulated in a conidiation temperature-dependent manner (Fig 4B). Both sets of genes were expressed at lower levels in the conidia from the 45°C culture, compared with those from the 37°C culture (S2 Table). One cluster contained four genes, including a gene encoding a predicted 1,4-alpha-glucosidase, whereas the other contained five genes, including *pr1*, which encodes a putative alkaline serine protease. The two sets of genes did not appear to be involved in secondary metabolism. This MIDDAS-M analysis confirmed the temperature-dependent transcriptional activation of the *tpc* cluster.

**Fig 5:**
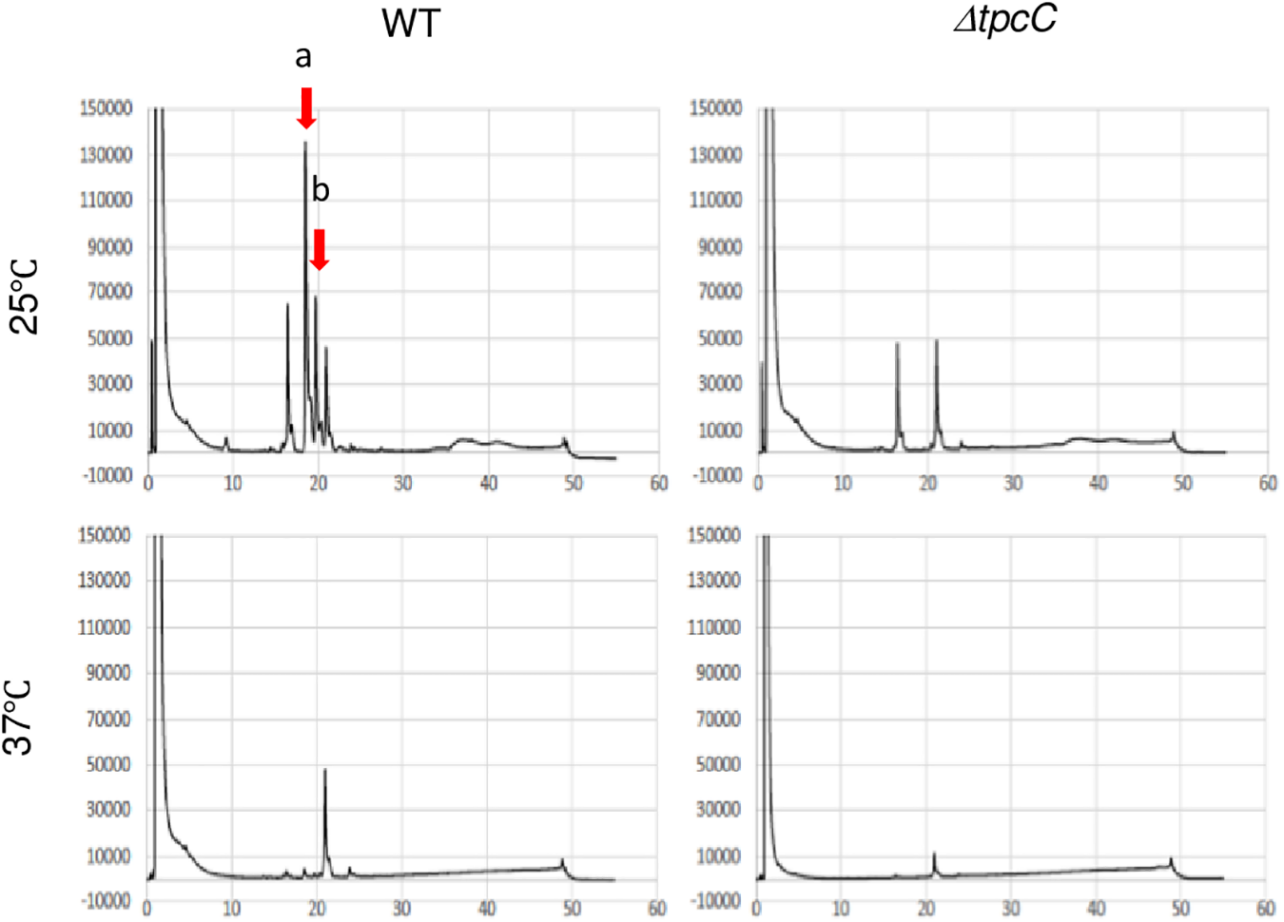
Chromatograms from the HPLC analysis of the WT and *ΔtpcC* strains. Samples were extracted from conidia that were harvested from 25 and 37°C cultures. The crude extract was injected into an HPLC Wakosil-II system with a 3C18 HG column, and it was monitored by a photodiode array detector. The chromatograms detected by the absorbance at 330 nm are shown. Arrows (a and b) indicate the peaks specific to WT conidia from the 25°C culture. Arrow a: trypacidin. Arrow b: monomethylsulochrin.

**Fig 6:**
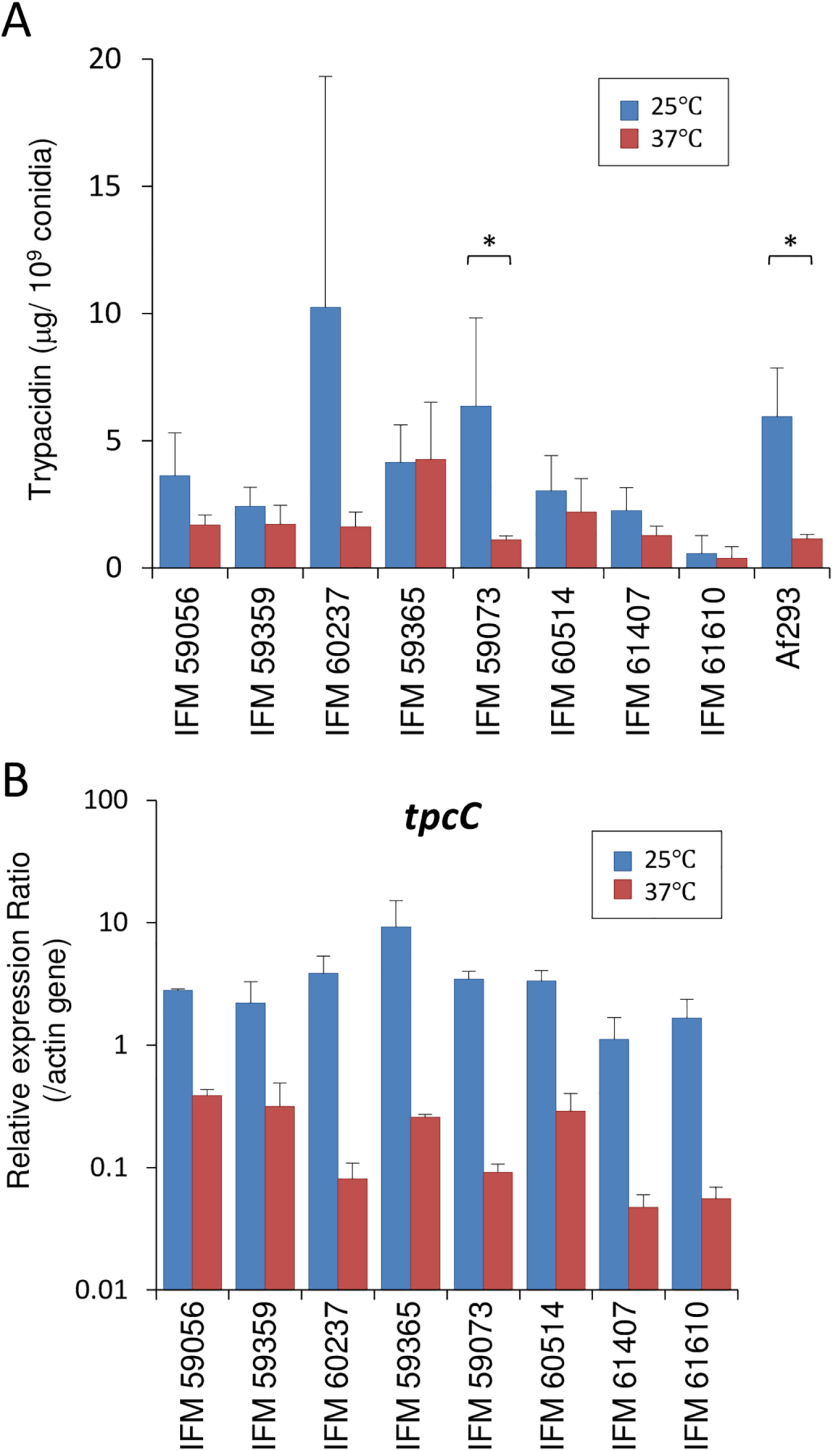
Quantification of trypacidin production in clinical isolates. (A) Samples were extracted from the conidia of clinical isolates as well as Af293 strains that were harvested from 25°C and 37°C cultures, and they were analyzed by HPLC. The amount of trypacidin was quantified using a standard curve. Each sample was tested in 3 to 5 replicates. Error bars represent the standard deviation. Significant differences (by Student’s *t*-test) between conidia from 25°C and 37°C cultures were indicated with asterisk (*: *p*<0.05). (B) Gene expression analysis of *tpcC* in the clinical isolates. The eight clinical isolates were grown on PDA for 7 d at 25 and 37°C. Then, conidia were harvested, and their RNA was extracted. The expression ratio of *tpcC* relative to actin was determined by qRT-PCR analysis. Error bars represent standard deviations based on three biological replicates.

To investigate whether trypacidin production was affected by the conidiation temperature, we analyzed extracts of conidia from a WT strain and a *tpcC* deletion mutant (designated *ΔtpcC*) that were cultured at 25 and 37°C (Fig 5). We found that two peaks in the Af293 conidia from the 25°C culture were not seen in the mutant or in the Af293 conidia from the 37°C culture. The compounds corresponding to peaks with retention times (RTs) of 18.5 and 19.7 min were isolated from the Af293 conidia from the 25°C culture. Their structures were identified by mass spectroscopy and a NMR analysis as trypacidin (RT: 18.5) and monomethylsulochrin (RT: 19.7). Monomethylsulochrin is a biosynthetic intermediate of trypacidin, and the production level of trypacidin was significantly lower in Af293 conidia from the 37°C culture compared with that in the conidia from the 25°C culture (Fig 6A). Thus, the *tpc* cluster was exclusively and highly activated at 25°C during conidiation.

### Temperature-dependent trypacidin and DHN-melanin production differ in clinical strains

To broaden our knowledge of the temperature-dependent accumulation of trypacidin, we investigated the ability of eight clinical strains, which were isolated from patients with chronic pulmonary aspergillosis, to produce trypacidin. Trypacidin quantification showed that the strains IFM 59073 and Af293 contained significantly higher amounts of trypacidin in the conidia harvested from 25°C cultures compared to those from 37°C cultures (Fig 6A). More interestingly, the IFM 59365 conidia accumulated abundant trypacidin, even when they were cultured at 37°C (Fig 6A). Then, the relative level of the *tpcC* transcript was determined in the conidia of the clinical isolates. All of the isolates showed higher transcript levels in the conidia from the 25°C cultures compared with those from the 37°C cultures (Fig 6B). Thus, trypacidin production was not correlated to the *tpcC* transcript level.

**Fig 7:**
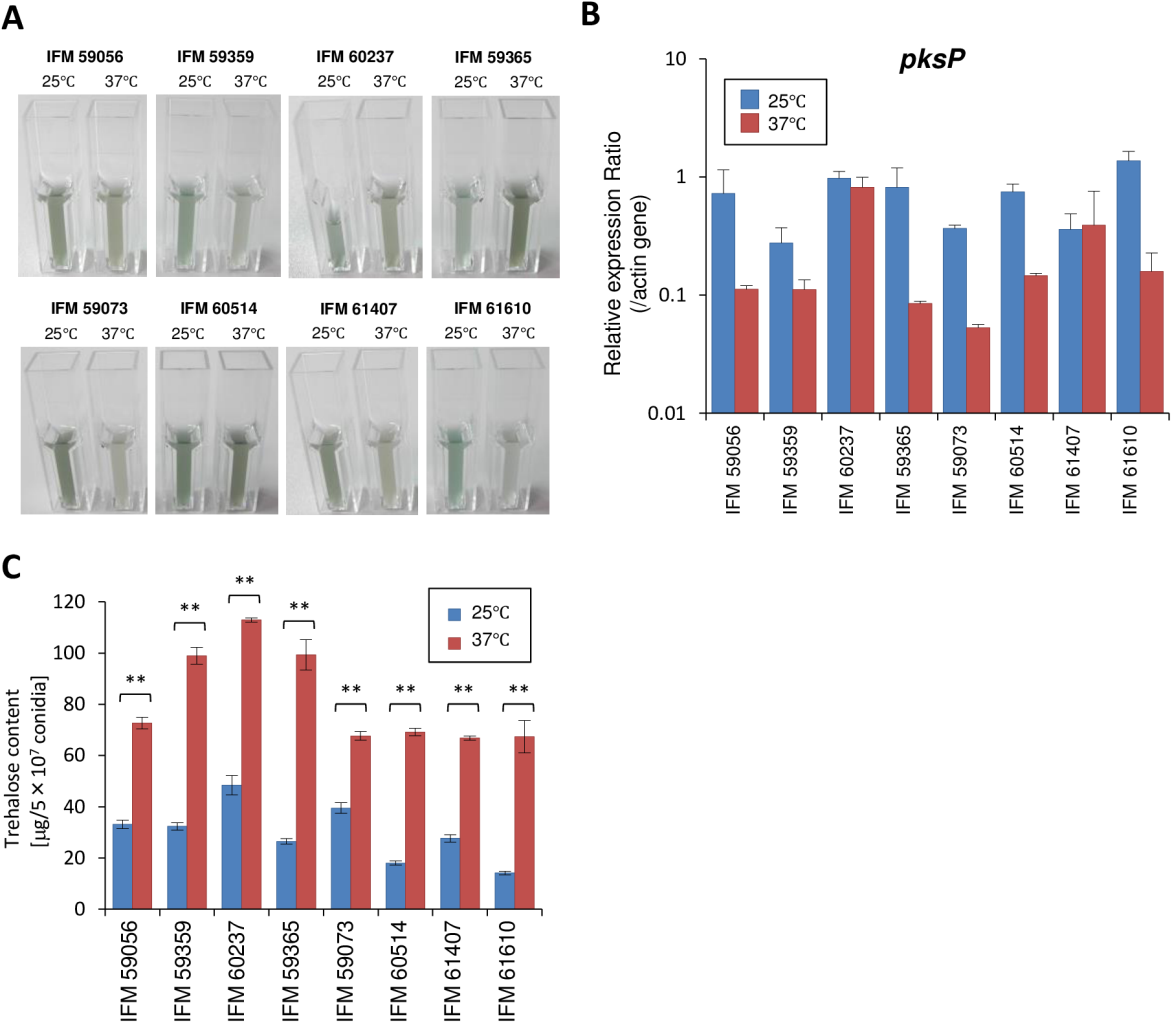
Effect of temperature on pigmentation in the clinical isolates. (A) Comparison of conidial pigmentation. Conidia of the clinical isolates were harvested from 25 and 37°C cultures. Conidial suspensions were diluted to 10^8^ conidia mL^−1^ and photographed. (B) Expression analysis of *pksP*. RNA was extracted from conidia that were produced on PDA at 25 and 37°C. The expression ratio of *pksP* was determined by qRT-PCR. Each sample was tested in three biological replicates. Error bars represent standard deviations. (C) Trehalose was extracted from the conidia of clinical isolates and quantified by glucose assay after trehalase digestion as described previously [5]. Error bars represent the standard deviation based on three independent replicates. Significant differences (by Student’s *t*-test) between conidia from 25°C and 37°C cultures were indicated with asterisk (**: *p*<0.01).

In addition to trypacidin, we determined the temperature-dependent regulation of DHN-melanin accumulation in clinical strains. Among these strains, IFM 59056, IFM 59359, IFM 59073, IFM 61407, and IFM 61610, showed deeper conidial pigmentation levels in the 25°C cultures than in the 37°C cultures (Fig 7A). In contrast, the IFM 59365 conidia from the 37°C culture exhibited a darker pigmentation compared with those from the 25°C culture, whereas no clear difference in pigmentation was seen in the conidia of IFM 60237 and IFM 60514 (Fig 7A). IFM 59365 accumulated both trypacidin and pigments in the conidia from the 37°C culture, which is suggestive of a link between the temperature-responsive mechanism regulating trypacidin and pigment production in *A*. *fumigatus* conidia. An analysis of the *pksP* transcription level revealed that all of the clinical strains, except IFM 60237 and IFM 61407, exhibited a higher level of the *pksP* transcript in the conidia from the 25°C cultures (Fig 7B). The experiments using clinical isolates showed that trypacidin and pigment production vary from strain to strain. Despite the strain-dependent temperature effects on trypacidin and pigment production, the effect on trehalose accumulation in the clinical isolates was largely similar to that observed in Af293, where the conidia from the 37°C culture contained more trehalose (Fig 7C).

## Discussion

In general, melanin contributes to the survival of fungal propagules in harsh environmental conditions [29]. Our results support the role of DHN-melanin in protection against UV irradiation in *A*. *fumigatus* conidia. The conidia from the 25°C culture were sensitive to heat and oxidative stress most probably because of a decreased trehalose content. One possible reason for this reduction is the increased intracellular trehalase activity. TreB is a neutral trehalase involved in trehalose breakdown during the germination stage [30], and the transcript level was relatively high in the conidia harvested from the 25°C culture (Fig 2B). Importantly, the temperature effects on conidial properties, such as pigmentation, trehalose accumulation, and heat stress tolerance, were also observed in the conidia harvested from cultures on glucose minimal medium (S1 Fig). Collectively, our results revealed that temperature has a great impact on the stress-resistance properties of conidia, which affects their survival in the environment.

DHN-melanin plays an important role in preventing the predation of *A*. *fumigatus* conidia by soil amoebae [14,31]. Trypacidin also affects the interaction between *A*. *fumigatus* conidia and amoebae [27]. Indeed, *tpcC* deletion mutant conidia were more sensitive to an amoebae challenge, which suggests that trypacidin is responsible for predation avoidance. Thus, it is tempting to assume that the fungus has evolved a temperature-dependent regulatory mechanism for melanin and trypacidin production to potentiate conidial survival in harsh environmental niches. Further studies will enable us to learn much more about how and why conidia are protected by secondary metabolites.

Trypacidin was initially determined to have an anti-protozoal activity [32]. Trypacidin is cytotoxic to A549 lung cancer cells and human bronchial epithelial cells, indicating that it is a potential mycotoxin [33]. Throckmorton *et al*. [26] showed that the A1163 strain is derived from a clinical isolate and did not produce trypacidin because of the genetic inactivation of the *tpcC* gene, indicating that the production of this mycotoxin is dispensable for the development of aspergillosis in a clinical setting. This view was also supported by virulence tests using a Toll-deficient *Drosophila* model [26], in which there was no significant difference in virulence between a *tpcC* mutant and a WT strain. During infection, conidia must germinate on and in the lung tissues. Thus, it is possible that trypacidin contributes to the injury of attached or surrounding cells, including epithelial cells and phagocytes, during germination. In swelling conidia, there was no increased expression of related genes, such as *tpcC*, *in vitro*, suggesting that there was no *de novo* trypacidin synthesis during the swelling/germinating stage (data not shown). Thus, trypacidin may particularly contribute to conidial protection, and it may not be a prerequisite for *A*. *fumigatus* virulence.

Our results highlighted the temperature effect on conidial properties, such as stress tolerance, and the accumulation of DHN-melanin and mycotoxin. This should be considered when conidia suspensions are prepared for phenotypic studies, including infection experiments. Different incubation temperatures might affect the results.

Another notable finding is that the isolates exhibited heterogeneous responses, in terms of pigmentation and trypacidin production, to environmental temperature. It is empirically acceptable that all of the isolates of identical species, *i*.*e*., *A*. *fumigatus*, possess a genetically diverse background and, consequently, exhibit different phenotypes, such as hyphal growth, conidiation, conidial pigmentation, nutrient preferences, and virulence [34,35]. The analysis of our set of clinical isolates revealed that some strains produced more densely pigmented conidia in the 25 or 37°C cultures, whereas others produced highly pigmented conidia equally at both temperatures. Because DHN-melanin protects conidia from UV-irradiation stress, highly pigmented conidia appear to be preferable for conidial survival under UV- or strong sunlight-exposure conditions. The fungus might have evolved the temperature-dependent regulation of stress protection in a strain-dependent manner to adapt to its environment. For a deeper understanding of conidia stress-resistance-related physiology, heterogeneity among isolates should be taken into account, and exploring the diversity of conidial properties may enable the identification of previously unknown stress-resistance factors.

In conclusion, we presented the first evidence that conidial stress resistance and secondary metabolism are affected by temperature during conidiation in *A*. *fumigatus*. The findings of a low temperature-dependent overproduction of DHN-melanin and trypacidin in conidia provide new insights into the conidial physiology of filamentous fungi and pave the way for understanding the temperature-dependent regulation of secondary metabolism.

## Material and methods

### Strains and growth conditions

*A*. *fumigatus* strains Af293 was used to generate the *ΔtpcC* strain. The fungal strains used in this study are listed in S4 Table. The clinical strains IFM 59056, IFM 59359, IFM 60237, IFM 59365, IFM 59073, IFM 60514, IFM 61407, and IFM 61610 were isolated from patients with chronic pulmonary aspergillosis from 2007 to 2012 in Japan, and their detailed descriptions were provided in a previous study [36]. All strains were cultivated in PDA at 25°C, 37°C, and 45°C for conidia collection. The cultivation time was 7 d, unless stated otherwise.

#### Construction of the *tpcC* gene deletion mutant

The mutants used in this study were constructed by gene replacement method that used a hygromycin B resistant marker (*hygB*^*r*^) in a double homologous recombination event. The deletion cassettes for each gene were constructed using the GeneArt Seamless Cloning and Assembly Kit (Invitrogen, Carlsbad, CA, USA). To create the deletion cassettes, 5′- and 3′-flanking regions of the target gene and the *hygB*^*r*^ fragment were fused and introduced into the pUC119 vector. The *hygB*^*r*^ fragment was amplified and obtained from pCB1004 by PCR using the primer pair hygBr-F and hygBr-R (S5 Table). To amplify fragments for cloning, KOD plus ver.2 high-fidelity DNA polymerase (Toyobo, Osaka, Japan) was used for all the PCRs.

To delete *tpcC* (Afu4g14560), 5′- and 3′-flanking regions were obtained by PCR using the primers tpcC-U-F(pUC119) and tpcC-U-R(hygBr), and tpcC-D-F(hygBr) and tpcC-D-R(pUC119) (S5 Table). These flanking regions and the *hygB*^*r*^ fragment were ligated into pUC119 using the GeneArt system, resulting in the plasmid, pUC-*tpcC*::*hygBr*, from which the cassette for transformation was amplified using primers tpcC-U-F and tpcC-D-R (S5 Table).

Transformation of *A*. *fumigatus* was performed according to a conventional protoplast-polyethylene glycol transformation method for *Aspergillus* [37]. Homologous recombination and correct gene replacement were confirmed by PCR of genomic DNA, and the absence of the transcript of the target gene was verified by RT-PCR analysis. We obtained at least three independent transformants for each gene deletion, and the data from a representative strain were presented.

#### Conidia preparation

Conidia of each strain were stored in 20% glycerol in a −80°C freezer. To prepare fresh conidia, the stored conidia were inoculated on a PDA slant and incubated at 37°C for 1 week. The conidia were harvested with phosphate-buffered saline (PBS) containing 0.1% Tween 20, and the concentration was calculated by counting the conidia with a hemocytometer (Watoson, Kobe, Japan). To collect conidia for RNA purification and trypacidin extraction, conidia were mixed with 15 mL of PDA (final concentration, approximately 10^4^ conidia/mL) before the medium solidified in a 100-mL flask, and they were incubated at 25°C, 37°C, or 45°C, for 7 d (or culture age as indicated) in the dark. After the cultivation, conidia were harvested with PBS-Tween 20, filtered by a Miracloth, counted with a hemocytometer, and washed once with PBS-Tween 20. Microscopy observations revealed that there were no hyphal fragment contaminants in the conidial suspension, which contained exclusively conidia.

#### Stress test for conidia

Viability assays for heat stress and oxidative stress were performed as described previously, with slight modifications [5]. Briefly, suspensions of 10^5^ conidia mL^−1^ were incubated at 60°C for 15 min (heat stress assay) or in the presence of 200 mM H_2_O_2_ for 15 min at room temperature (oxidative stress assay). After stress treatment, the suspensions were immediately cooled on ice and diluted to 10^3^ conidia mL^−1^. To count colony-forming units (CFU), the suspensions (100μL) were spread on 0.1% yeast extract-containing glucose minimal medium plates. Conidia without stress treatment were used as a reference, and survival rates were calculated in three or four technical replicates. CFUs were counted from the plates in each test.

For the UV exposure test, 100 conidia were spread onto 0.1% yeast extract-containing glucose minimal medium plates, and the plates were exposed to 9.55 W m^−2^ of UV light for 1 min, providing a dose of 573 J m^−2^. After UV exposure, the plates were incubated at 37°C in the dark for more than 24 h before counting the CFU. The survival rate was calculated by dividing the CFU obtained following UV exposure by the CFU obtained in the absence of UV exposure. CFUs were counted from five plates.

#### Polyols and trehalose quantification

Polyols and trehalose contents in conidia were measured as described previously [5]. Briefly, conidia harvested from each culture condition were suspended in H_2_O and incubated at 98°C for 3 h to extract the polyols and trehalose. After filtration with an Amicon Ultra concentrator (Millipore, Billerica, MA, USA), the contents were quantified by high-performance liquid chromatography using a Shodex Asahipack NH2P-50 4E column. The mean value was obtained from six biological replicates. Trehalose content was also determined by glucose quantification after degrading by trehalase as described previously [5].

#### Melanin test

To compare conidial pigmentations, suspensions of freshly harvested conidia were prepared and diluted to 10^8^ conidia mL^−1^. The pigmentation levels of the conidial suspensions were visually compared in a cuvette. TCZ was used to inhibit the DHN-melanin synthesis pathway [24]. TCZ was added to PDA at a final concentration of 10 μg mL^−1^. Preliminary experiments confirmed that 1 μg mL^−1^ was insufficient to repress the production of DHN-melanin, and that 10 μg mL^−1^ of TCZ did not affect colony growth or conidial production levels.

#### RNA and cDNA preparation

Mycelia and conidia were harvested, washed with distilled water, and frozen in liquid nitrogen, and total RNA was isolated using the FastRNA Pro Red Kit (MP Biomedicals, Santa Ana, CA, USA). For quantitative real-time RT-PCR analysis, to obtain cDNA pools from the total RNA, removal of possible contaminating genomic DNA and reverse transcription were performed using the ReverTra Ace qPCR RT Master Mix with gDNA remover (Toyobo).

#### RNA sequencing analysis

For RNA-sequencing analysis, sample libraries were prepared from total RNA by the TruSeq RNA Sample Prep Kit v2 (Illumina, San Diego, CA, USA) according to standard protocols and as performed previously [5]. Briefly, each total RNA sample (1 μg) was enriched for mRNA using oligo (dT)-tagged beads. The mRNA samples were fragmented into smaller pieces and used to synthesize cDNAs. The library construction involved end repair, A-tailing, adapter ligation, and amplification. The mean length for each library was approximately 280 bp. Sequencing was performed in a single-end 50 base pair mode on a Miseq system (Illumina).

To compare the expression levels of each gene, the sequences were analyzed using a CLC Genomics Workbench (CLC Bio, Aarhus, Denmark). The sequence reads were trimmed and the quality was checked. Only reads with quality values higher than Q30 (approximately 97.2% of the total reads) were used for mapping. The *A*. *fumigatus* Af293 genome data, which was retrieved from the National Center for Biotechnology Information (http://www.ncbi.nlm.nih.gov/bioproject/PRJNA14003), was used as the template for mapping. From the mapping data, FPKM values were calculated using a program in the CLC Genomics Workbench. The RNA-sequencing read data was deposited to the DDBJ Sequence Read Archive (http://trace.ddbj.nig.ac.jp/dra/index_e.html) under accession No. PRJDB5526.

#### MIDDAS-M analysis

The gene clusters whose transcript levels are coordinately changed in the conidia cultured at 25°C or 45°C compared to those at 37°C were investigated using the MIDDAS-M algorithm [28]. Briefly, the induction ratio of genes at 25°C or 45°C over 37°C were evaluated in the logarithmic form with the base of 2 using the transcriptome data. After the Z-score normalization, the gene cluster expression score were calculated for each gene using the algorithm. The maximum cluster size was set as 30. By setting the threshold of the score as 0.05, candidate clusters were detected. The threshold values are respectively 8743 and 24358 in the 25°C/37°C and 45°C/37°C datasets.

#### Quantitative real-time RT-PCR

Real-time RT-PCR was performed using SYBR Green detection as described previously [37]. The primer sets used in this study are listed in S5 Table. The actin gene was used as a normalization reference (internal control), and the relative expression ratio to actin gene was calculated. Each sample was tested in three biological replicates.

#### Extraction and chemical analysis

Conidia (10^9^) from each strain were suspended in 100 μL of PBS and ethyl acetate (1:9) and were homogenized using a MagNA Lyser Green Beads tube (Roche, Basel, Switzerland). After centrifugation, the supernatant, consisting of the organic phase, was collected and dried *in vacuo*. The crude extract was dissolved in 100 μL of dimethyl sulfoxide (DMSO). Samples were injected into a high-performance liquid chromatography (HPLC) Wakosil-II system with a 3C18 HG column (particle size: 3μm; length: 150mm; internal diameter: 3mm) (Wako Pure Chemical Industries, Osaka, Japan). The column was run using a gradient of 10–100% acetonitrile in water over 45 min at a flow rate of 1 mL min^−1^ and monitored by a photodiode array detector (SPD-M10A VP, Shimadzu Corp., Kyoto, Japan). To quantify trypacidin production, peak areas detected by the absorbance at 330 nm were obtained in 3 to 5 biological replicates. A trypacidin standard was purchased from AdipoGen (San Diego, CA, USA) and used to create a standard curve.

To identify chemical structures, peaks corresponding to trypacidin and monomethylsulochrin were fractionated and approximately 1 mg was collected. Their structures were identified from the following spectroscopic parameters.

Trypacidin: ^1^H NMR (500 MHz, CDCl_3_): **δ** = 2.42 (s, 3H), 3.64 (s, 3H), 3.67 (s, 3H), 3.93 (s, 3H), 5.75 (d, *J* = 1.1 MHz), 6.35 (s), 6.53 (s), 7.08 (d, *J* = 1.1 MHz); ^13^C NMR (125 MHz, CDCl_3_): **δ** = 23.4, 53.0, 56.3, 56.9, 84.3, 104.2, 105.6, 105.8, 108.6, 137.3, 138.5, 152.3, 158.6, 163.7, 169.7, 175.0, 185.9, 190.7; HR-ESI-MS: *m*/*z* 345.0976 [M+H]^+^, calcd. for C_18_H_17_O_7_: 345.0974.

Monomethylsulochrin: ^1^H NMR (500 MHz, CDCl_3_): **δ** = 2.27 (s, 3H), 3.35 (3H), 3.67 (s, 3H), 3.69 (s, 3H), 6.04 (s), 6.44 (s), 6.60 (d, *J* = 2.3 MHz), 7.01 (d, *J* = 2.3 MHz); ^13^C NMR (125 MHz, CDCl_3_): **δ** = 22.7, 52.4, 55.9, 56.5, 103.1, 103.4, 108.0, 110.0, 110.7, 128.5, 128.7, 148.2, 156.3, 157. 4, 161.1, 164.5, 166.3, 199.6; HR-ESI-MS: *m*/*z* 369.0948 [M+Na]^+^, calcd. for C_18_H_18_O_7_Na: 369.0947.

## Supporting information

S1 Fig Temperature effects found in the conidia from cultures on glucose minimal media (GMM). (PPTX)

S1 Table FPKM list for 8953 genes. (XLSX)

S2 Table FPKM list for the allergenic genes. (XLSX)

S3 Table FPKM list for the genes that were coordinately regulated. (XLSX)

S4 Table Strains used in this study. (XLSX)

S5 Table PCR primers used in this study. (XLSX)
