# Supplementary material for: Temperature during conidiation affects stress tolerance, pigmentation, and trypacidin accumulation in the conidia of the airborne pathogen Aspergillus fumigatus
Source: PLoS One. 2017 May 9;12(5):e0177050. doi: 10.1371/journal.pone.0177050 (PMC5423626; doi:10.1371/journal.pone.0177050)
Supplement: S1 Fig — (PPTX) [file pone.0177050.s001.pptx]

## Slide 1
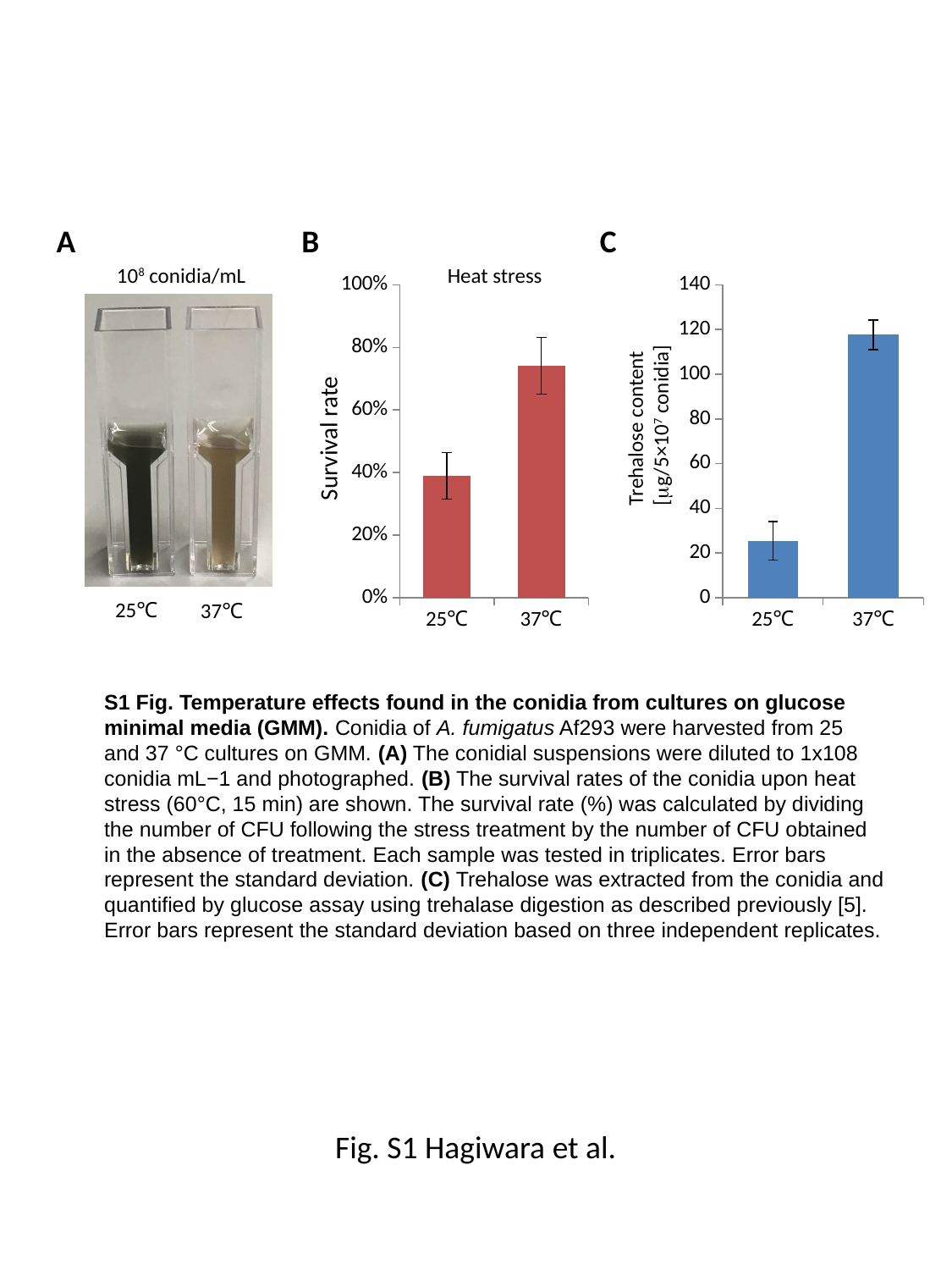

A
B
C
108 conidia/mL
Heat stress
### Chart
| Category | |
|---|---|
| 25℃ | 0.389473684210526 |
| 37℃ | 0.741007194244604 |
### Chart
| Category | |
|---|---|
| 25℃ | 25.5 |
| 37℃ | 117.6666666666667 |
Trehalose content
[mg/5×107 conidia]
Survival rate
25℃
37℃
S1 Fig. Temperature effects found in the conidia from cultures on glucose minimal media (GMM). Conidia of A. fumigatus Af293 were harvested from 25 and 37 °C cultures on GMM. (A) The conidial suspensions were diluted to 1x10^8^ conidia mL−1 and photographed. (B) The survival rates of the conidia upon heat stress (60°C, 15 min) are shown. The survival rate (%) was calculated by dividing the number of CFU following the stress treatment by the number of CFU obtained in the absence of treatment. Each sample was tested in triplicates. Error bars represent the standard deviation. (C) Trehalose was extracted from the conidia and quantified by glucose assay using trehalase digestion as described previously [5]. Error bars represent the standard deviation based on three independent replicates.
Fig. S1 Hagiwara et al.
